# Taking the responsibility in dementia care: A concept analysis about facticity

**DOI:** 10.1002/nop2.162

**Published:** 2018-06-01

**Authors:** Célia Pereira Caldas, Carina Berterö

**Affiliations:** ^1^ Nursing Faculty Rio de Janeiro State University Rio de Janeiro Brazil; ^2^ Division of Nursing Science Department of Medical and Health Sciences Linköping University Linköping Sweden

**Keywords:** concept analysis, dementia care, facticity, nursing practice, philosophy

## Abstract

**Aim:**

The aim of this study is to develop a comprehensive definition of facticity, applicable to dementia nursing.

**Methods:**

Walker and Avant's Concept Analysis was used to analyse facticity. Published literature found in PubMed, CINAHL, PsycInfo, and Scopus using the search term facticity and nursing, as well as books and dictionaries and empirical data was used to clarify the concept.

**Results:**

Facticity in nursing dementia care is being in reality taking on responsibility to truth. Antecedents of facticity include persons occupied with caring a demented older person including full‐time duties, often accompanied by resistive behaviours. They are overloaded both physically and psychologically. Being present and feeling compassion and nurturing the relationship with the demented older persons and in that way strengthen the caregiving process.

**Conclusion:**

Nurses need to be aware that all human beings are living with facticity.

**Why is this research or review needed?**

- Caring a demented older person includes full‐time duties, assisting in care and other activities of daily living.
- Assistance with these care needs of the person with dementia is often accompanied by resistive behaviours.
- Comprehending facticity enhances the understanding of resistive behaviours.

**What are the key findings?**

- Facticity is useful in dementia care; nurses and healthcare providers may use creativity searching for understanding about how patients and next of kin perceive facticity.
- Facticity is being in reality, taking on responsibility to truth and having the ability to understand meaning and show affective willingness in our experience of the world.

**How should the findings be used to influence policy/practice/research/education?**

- When the nurse is open to the opportunities of integral care, she can promote connections between the different realities: nurse–patient and next of kin.
- Understanding facticity strengthens the understanding of our realities and thereby also improves the quality of care.
- Nurses need to be aware that all human beings are not just living in reality, but with facticity.

## 1 INTRODUCTION

It might be argued that facticity is only understood and used by professionals in the area of philosophy. However, phenomenological studies by nurses of nurses' and patients' experiences of lived experiences show an understanding and use of the concept of facticity (Lindberg, Horberg, Persson, & Ekebergh, 2013; Moreira & Sales, 2010; Oliveira & Menezes, 2014; Sebold, Kempfer, Girondi, & Prado, 2016), even though they do not verbally use the term facticity. Nurses are often intuitively acting and re‐acting without naming the responses—silent knowledge. All caregivers', no matter professional caregiver or next of kin, presents different responses to the obligation to provide care and make themselves accountable to themselves and others for what they do and say about caring the demented person. Putting words on what they really are doing in their care. Caring as unveiling the meaning of being can contribute to professional practice to the extent that this knowledge leads to less interventionist and more comprehensive approaches facing a highly complex problem, that makes it very vulnerable to stress situations (Heidegger, 1962/1997). Moreover, this understanding will enable forwarding and suggesting authentic caring approaches aiming at quality not only for the elders, but also for all kinds of caregivers, no matter if professionals or family, as long as all kind of support is needed to meet the older people needs. Bonevac (2014) point out the Heideggerian view of care as two forms of care. First, the inauthentic care which encourages and produces dependence between the ill person and the nurse or caregiver. The second form of care encourages and produces, as much as possible, independence. These aspects of care and being authentic are related to facticity.

The notion of understanding facticity as a component of dementia care and giving it practical attention is very important. Caring a demented older person includes full‐time duties, fulfilling universal needs of daily living. Assistance with these care needs of the person with dementia is often accompanied by resistive behaviours. Providing physical assistance and activities of daily life, make the caregivers to be confronted with concurrent resistiveness to care. These duties lead to physical and psychological overload (Braungart, Femiab, & Zarit, 2016) and can lead to depression. It has been shown that caregivers who had greater compassion reported more intrusive thoughts even when perceived physical suffering of the demented older person was low. Being a highly compassionate caregiver has negative effects, although higher levels of caregiver compassion likely reflect a high‐quality relationship with the demented older person and is an important factor in the caregiving process (Schulz, Savla, Czaja, & Monin, 2016).

Estimated figures suggest that between 40% and 70% of caregivers have clinically significant symptoms of depression, with approximately one fourth to one half of these caregivers meeting the diagnostic criteria for major depression (Riedel, Klotsche, & Wittchen, 2016; Zarit, 2006). Caldas and Berterö (2012) add that in the caring encounter, a hard management of temporality can lead the nurses and healthcare providers to burnout and/or a feeling of lack of accomplishment. Burnout can also lead to cynicism and to a distant attitude toward people and one's work (Berterö, 1999).

The quality of care is related to the quality of caregivers' relationship. Silverstein and Giarrusso (2010) published a review on studies of ageing families and found that dynamic models are essential for understanding how and why families and relationships change over time. They concluded that family caregiving is best understood as a career, with early family antecedents, cyclical involvements, and short‐ and long‐term impacts. The obligation to take care of older people cannot be based on a supposition of a good relationship between generations. It is clear that this supposition can easily be challenged by the generation conflicts and disagreements as a consequence of incompatible personalities, values and life styles of the young and older people. However, although the older people traditionally expect to be cared for by the young generation and professionals and children have no doubt about their responsibilities, these expectations have been changing.

Traditional responsibilities' have been changing as a consequence of social changes. The greatest influence of urbanization is certainly the change from the big family structure to the nuclear one, which reduce the relative's responsibilities towards the elder. On the other hand, young people live less and less together with the older generations in our modern society (Silverstein & Giarrusso, 2010).

### 1.1 Literature review

The literature search performed in PubMed, CINAHL, PsycInfo, and Scopus using the search term facticity and nursing resulted in some hits in philosophy and phenomenology. The combination of the search terms facticity and nursing resulted in one article in English, a discussion paper about epistemology.

The word facticity is defined in Webster's dictionary (Merriam‐Webster On Line Dictionary, 2017) as “the quality or state of being a fact.” Facticity has its origin from French facticité, from German faktizität, from Classical Latin *factum*, fact. Facticity could be referred to facts, factuality, or reality, but for Dilthey, according to Palmer (1969), it means that which resists explanation and interpretation. Man was seen as a historical being, but not in term of an object of the past, but “a series of world views.” Man can only understand himself through the lived experience, which springs up out of the depths of his own being. Man's understanding is dependent on past worldviews, interpretations, and a shared world (Palmer, 1969). For Sartre (1969), facticity signifies all of the concrete details against the background of which human freedom exists and is limited. For example, these may include the family, the historical period, and the country where we were born, as well as the inevitable prospect of our death. There is a necessary connection with the person itself being in the world and its own past. This is in great agreement with what Heidegger designates facticity persons' experiences of a world of meanings and relations (Dahlstrom, 2013).

Heidegger (1962/1997) considers facticity as the “thrownness” (Geworfenheit) of individual existence. We are “thrown into the world.” By this, facticity is something that already informs and has been taken up in existence, even if it is unnoticed or left unattended. As such, facticity is not something we come across and directly behold. In moods, for example, facticity has an enigmatic appearance, which involves both turning toward and away from it. For Heidegger, moods are conditions of thinking and willing to, which they must in some way respond. The thrownness of human existence (or Dasein) is accordingly disclosed through moods.

Heidegger (1962/1997) defines facticity as being something, being in reality, being in the world, find him/herself bound up in its destiny, thrown in the world. Facticity is the way which every Being actually is. Facticity is the Being's mode of contingent existence. Authentic retrieval of facticity is a response to my obligation to make myself accountable to myself and others for what I do and say. Facticity is taking one's responsibility to truth.

The Being can be opened or closed in his thrownness (being released in the world). Being opened means to be aware of being released and take responsibility for one's own decisions, accounting for their weight. The thrownness of human existence is accordingly disclosed by moods. The Being is always marked by understanding (ability to assign meaning) and all understanding is affectively toned. Understanding and affective willingness (moods) are coconstitutive of the Being. Both the willingness and understanding have the ability to open up the Being.

Discussing the essence of nursing practice, Kim Hesook (2015) detaches three principles of existential nursing rationality. The first principle is authenticity that self‐making involves a burden of choice requiring one to be true to oneself, by proper coordination between transcendence and facticity. There is responsibility taken of choices that are made. Since one's being is a complex of facticity and transcendent entirety, “doings” reflecting choices are governed by how authentic one is in coordinating this “being.” The existential character of nursing practice revealed in nursing comportment governed by authenticity is in the genuine “caring.” The second principle is particularism focusing on professional services as nursing practice. Particularism in the existential sense is associated with the principle of authenticity, but goes deeper to point out the need of particularity of each “doing.” The third principle is understanding, but not the cognitive one. This is the understanding of oneself and a situation. It is about grasping the meanings in a holistic way. These three principles frame the existential dimension of nursing practice and refers to how nurses' comportments are made to be meaningful and significant as nursing situations (Kim Hesook, 2015). The aim of this study is to develop a comprehensive definition of facticity, applicable to dementia nursing.

### 1.2 Concept analysis

To get a broader perspective about the phenomenon, the framework for concept analysis was used when examining the concept of facticity. This method is the most frequently used in nursing science and it is one of the first one that theoretically discuss concepts of the nursing sciences area (Duncan, Duff Cloutier, & Bailey, 2007). This method has been criticized and modified but it is still used, many studies are applying it.

Using concept analysis by Walker and Avant, demand senior nursing researchers with good competence in philosophy and in the area of research (Bergdahl & Berterö, 2016). The same authors state that important concepts can have many possible meanings and can be used in different ways in a discipline. That is why the present paper is aiming for bringing forth the use of a philosophical concept applied in nursing dementia care.

This analysis is based on philosophical, theoretical, and scientific literature, working with the Walker and Avant method, which is presented in eight steps:

- First, a concept should be selected. Descriptions of facticity or similar terms were found in the literature to be presented with different perspectives. Searches in several databases produced no concept analysis of facticity. Deeper understanding of facticity may enhance dementia care in various caring encounters.
- Second, the purpose of analysis should be determined. The purpose of this concept analysis is to develop a comprehensive definition of facticity applying to dementia care.
- Third, identify all uses of the concept that can be discovered. These uses are presented in the results.
- Fourth, determine the defining attributes.
- Fifth, identify and present a model case.
- Sixth, identify borderline/related and contrary cases.
- Seventh, identify antecedents and consequences.
- Finally, the eight step is to define empirical referents (Walker & Avant, 1995; Walker & Avant, 2005).

### 1.3 Data analysis

The data analysis was conducted by investigating how the data from the literature did present the concept. An organized reading of all data materials was done to identify the defining attributes of the concept facticity. Cases were identified based on the defining attributes and antecedents from the authors nursing practice; consequences and empirical referents were identified based on the philosophy of Heidegger and on its relationship with the defining attributes.

### 1.4 Ethics

Ethical approval was not required since this is a philosophical and theoretical paper applied on clinical practice.

## 2 RESULTS

### 2.1 Attributes

Based on the available literature, dictionary definitions of *facticity* and the various usages of the concept in the disciplines of philosophy and nursing, include the following attributes. Facticity could be seen as being in reality and taking on responsibility to truth (Heidegger, 1962/1997; Kim Hesook, 2015). Facticity is also about ability to understand meaning and an affective willingness (Heidegger, 1962/1997). It is a central component of our experience of the world (Dahlstrom, 2013; Sartre, 1969). The person who is in facticity is in that person's reality influenced by past and present experiences as well as the context. The person understands the meaning of the situation and takes on responsibilities to be truthful to herself and to others.

### 2.2 Model case

A model case is an example of the use of the concept that demonstrates all the defining attributes of the concept (Walker & Avant, 2005). An example of a model case would be the case of Rose.

Rose is the main caregiver of her father. He has Alzheimer dementia and Rose is always challenged for his dependence and sometimes, disruptive behaviour. She has a brother and two sisters. Rose is the youngest, but she has always assumed responsibility for the whole family whatever the issue has been caring for someone. Her brother only visits the father twice a year and he is the one who had the best relationship with the father. In the beginning, Rose's sisters did avoid assisting Rose in her caring efforts.

After some time of reflection and coping, Rose realized that the situation she is in, also exactly is what she has to live. So, she decides to live the beauty and also the difficulties of each day, one day at a time. She decides to be present and take the responsibility of her father's care but without denying her own opportunities of having a life. Through compassion, she has been nurturing the relationship with her father. It was important in this process, to have support of Nurse Karen, the geriatric nurse who is advising Rose at the Health Centre. Together, caregiver and nurse have found the way to strengthen the caregiving process.

Nowadays, Rose's sisters participate with some care tasks and also when Rose needs to rest or take care of her own life. Rose thinks that helping her father is the obvious thing to do. It is not a sacrifice. Sometimes, she is overloaded both physically and psychologically and sometimes she feels anger and anguish. But now she knows that there is support from her sisters and there is support and advice of Nurse Karen.

### 2.3 Borderline case

Walker and Avant (2005) define related/borderline cases as instances of concepts that are related to the concept being studied but do not contain all the defining attributes. It is the case of Jennifer, the daughter of Dora. She renounced her own life to take care of her mother. She lives through her. Jennifer no longer cares about her husband and children. The only focus Jennifer has is the responsibility for her mother. She feels overwhelmed by the high degree of dependence of her mother. She feels that her mother's illness is “killing” her, that is, taking care of her mother is taking away her health. By dedicating herself completely to her mother, she is left without care for herself. She says that taking care of her mother is patience and renunciation. Jennifer does not let anything fail or lack regarding good care of her mother, because she knows law obliges her. But Jennifer constantly says this is not fair. The government should take care of the older people, because her mother is retired and have paid social security for her whole lifetime.

Miriam, a specialized geriatric nurse, tried to advise Jennifer, but she did not succeed. It is very difficult for her to understand that with some creativity, patience, and compassion, it would be possible to give good care to Dora, without renounce to her own opportunities of having a healthy and meaningful life.

### 2.4 Contradictory case

A contrary case is an example of “not the concept.” The case of Mary is one example of this not being the concept. Her mother Tina had advanced dementia. Mary and her brother Tony decided that they would not take care of her, even though Tina was not able to live by herself at her own home. They acknowledged that Tina was a good mother, but they had their own responsibilities with their own families and work. They found a very good nursing home and they trusted the professionals to take good care of their mother. When asked about why Mary did not visit her mother last Christmas, she answered that it was not possible because she had guests at home. Visiting her mother did not make sense anymore, since she did not remember who she is. Mary is confident that everything is all right because she asked the staff to call her when her mother is dead. She knows she is doing the right thing because the nurses supported her to do this way. That is why she is very satisfied with this nursing home.

### 2.5 Antecedents and consequences

Antecedents are events or incidents that occur before the occurrence of the concept (Walker & Avant, 2005). The antecedents are the predisposing factors that occur before the perception of facticity. Antecedents of the concept of facticity include persons occupied with caring a demented older person including full‐time duties, often accompanied by resistive behaviours. They are overloaded both physically and psychologically, which causes anger, anguish, and/or depression. One way they cope with this is to distance themselves from others, or even to get “burned out” (Berterö, 1999; Caldas & Berterö, 2012). The other way to cope is to stop and reflect, to realize that they are here and now‐they are being in the world, that is facticity. Being present and feeling compassion and nurturing the relationship (Kim Hesook, 2015) with the demented older person and in that way strengthen the caregiving process.

Walker and Avant (2005) define consequences as those events or incidents that occur as a result of the occurrence of a concept. When the care is given by the nurses, they are caring for a dependent person—the demented older person. The caring situation is here and now, but there could be difficulties in caring about the demented older person as the existential person they are—an acting being, with particular history and memories (Kim Hesook, 2015). If the care is given by the relatives, they are suffering since the person they are caring for is not the same person they knew, before being dependent. Both the nurses and informal caregivers are living in the world as “a series of world views,” bringing forth experiences, interpretations, and a shared world. The demented older person is living in another world, with limited world views, limited access to history, and memories (Dahlstrom, 2013; Heidegger, 1962/1997; Sartre, 1969). There are great challenges of caring for and about a demented older person when the personality and behaviour are changed. Caregivers, both informal and formal, should meet these challenges by using creativity, patience, and compassion.

### 2.6 Empirical referents

The final step in concept analysis is to identify the empirical referents that show how the concept is measured in the real world (Walker & Avant, 2005). It is difficult to measure facticity. As a matter of fact, facticity cannot be measured. It can be perceived. Facticity is the different life situations we see ourselves and others. Considering Heidegger(1962/1997) facticity as the “throwness”, whereas we as human beings are embedded in the world and basic life is not to be controlled by the person. What happens during life is a normal part of human existence. Facticity as the “throwness” means that we are exposed to different life situations. During these situations, human beings sometimes attend, sometimes letting things go, and sometimes giving up something. These ways of being are fundamental. Refusal to accept the facticity of life, for example, when having dementia or caring for a person with dementia could harm the life they are living as well as the obligation to make themselves accountable to themselves and others.

## 3 DISCUSSION

The notion of understanding facticity as a component of dementia care and giving it practical attention is very important. As nurses and healthcare providers, we need to realize that caring involves a burden of choice requiring one to be true to oneself. The genuine “caring” is to take responsibility of choices that are made. Nurses must take this responsibility and also understand how hard this issue is for the informal caregiver; often excluding the cognitive understanding. The informal caregivers are in need of existential understanding for themselves and their situation. That is what Kim Hesook (2015) reinforces when she mentions that nurses should “grasp the meanings in a holistic way.” It is about how nurses' comportments are made to be meaningful and significant as nursing situations.

The informal caregivers and the healthcare providers need to understand that despite all efforts to attend the requirements of caring activities, even though there is a 24‐hr day care, the patients' needs may not be met. Considering these facts, it is certain that existential facticity is lacking. There are some studies showing the importance of nurses going beyond the vision of reality and get the existential facticity understanding, even though it is not explicit stated.

Studying the impact of dementia on the demented person's relatives, Albinsson and Strang (2003) found that for the relatives, dementia is long‐lasting and devastating. The patient's gradual disappearance is especially stressful. The loss of verbal communication and, at the end, also a total absence of physical communication, makes the patient a living dead person before death actually occurs. Visiting the person with dementia is an obligation and not a free choice. There are feelings of guilt for placing the person at an institution, but also due to the staff, because failing the patient is not permitted. Actions had been taken by the institution, which is damaging the trueness of the next of kin. Factual life is affected. It was hard to see the patient. It is to be in a factual situation against ones will. In that case, guilt is about authenticity. Nursing action with these relatives is of great importance. Nurses have the opportunity to help them understand that being there with the demented person is not equivalent to living at the same place and taking care of him/her 24 hr a day. Being with the person is providing good care, even if it is professional care. The most important aspect in this caring situation is participating. Being participating, as a next of kin involved with the demented person, with the professionals and with the institution. Being is about a presence when visiting the demented person and not an obligation.

Shim, Barroso, Gilliss, and Davis (2013) studied the meaning in caring for a spouse with dementia in an institutional setting. Strategies such as accepting the situation, choosing a positive attitude, focusing on the blessings, or actively seeking resources could be helpful to any caregiver. Altruistic values, having the discipline to live those values, faith, and love may be important characteristics to human beings. Those attitudes among caregivers enhanced their ability to make their caring experiences more meaningful or positive.

Facticity has nothing to do with valuing only the good caregiving feelings. Che, Yeh, and Wu (2006) researched the empowerment process of primary caregivers and found that negative caregiving feelings not only inspired caregiver self‐awareness, but also cultivated their power to get rid of or even gain control over situations that they were struggling with. This power made the caregivers overcame their limitations and they became more positive and independent during times of difficulties.

Clissett, Porock, Harwood, and Gladman (2013) studied the challenges of achieving person‐centred care in acute hospitals. Person‐centred care means being there for the patient/the person with illness. They found that healthcare professionals were not making the most of every opportunity to make every intervention person‐centred. The result of this is that acute hospitals remain potentially harmful places for people with dementia. Edberg and Edfors (2008) add that the difficulties experienced by the nurses related mainly to the residents' behaviour. The authors detached that when the nurses stopped their tasks to reflect on the meaning behind the residents' behaviour, this, in turn guided their own actions; seeing the opportunities in their work. This insight may enhance facticity.

Eggers, Norberg, and Ekman (2005), studied fragmentation in the care of people with dementia. Their findings showed that care providers could counteract fragmentation by a caring based on attentive interest in the interaction with the person with dementia. By valuing the person behind the dementia disease, using an individual perspective considering the impact of the dementia disease and striving for mutual interpretation of the shared situation, it will enhance the patients to keep their world together. This could be referred to as facticity— being in reality and taking on responsibility to truth.

Eriksson and Saveman (2002) studied nurses' experiences of abusive/nonabusive caring for demented patients in acute care settings. The results showed that the nurses experienced various difficulties in meeting patients with dementia, which caused frustration. Those problematic situations could sometimes lead to abuse and neglect of these patients.

Facticity is the different life situations we see others and ourselves. Heidegger's (1962/1997) facticity as the “throwness” means that we are exposed to different life situations. During these situations, human beings interact in different ways.

According to a study by Acton and Miller (2003), a connected relationship with a higher power through a process that involved communication and discovery. There was a search for finding internal and external resources that facilitated comfort, peace, acceptance, and purpose in the caregivers' lives. When nurses integrate spiritual care, there is a opportunity of helping the connection with the patients or their next of kin. This is a state of being in facticity. The nurses stated that in these experiences, they saw caring in its entirety; this was good caring. This is in agreement with Heidegger (1923/1999), meaning that the being is to be understood in its own manner, as an entity in the world.

The caregiver who shows up as a person, who has to care for the person with dementia because there is no way out, is revealed as being‐there attached to facticity. The facticity can be explained from it to the extent that it has the ability to understand and interpret. That is, in opening up to understand that their situation is factual, the caregiver opens up him/herself to the opportunity of being free from their factual situation.

The factual character of being‐there is what each being‐there always is. That is, through facticity one can understand that in his “destiny,” that situation is linked to the being of that entity that comes to him/her in his own world. Anyway, it is what caregivers have shown as a “dead end” situation (Heidegger, 1962/1997). These ways of being are fundamentally related because they are issues that concern us.

Being is in itself, essentially, to be‐with. The caregiver has shown to be‐with the older people with dementia. It has been re‐emphasized that living with the person with dementia deems existentially the being that cares, even when the older person no longer communicates. The lack and absence of the person that the older person was in the past opens the caregiver to the opportunity of caring for the older person leaving the mere occupation and moving to a preoccupation. Even if the concern that the caregiver maintains is a deficient way, like being for the person, doing everything for him/her, it could also be not letting him/her decide by themselves anymore.

In contrast, the caregiver retains the opportunity of developing towards one's self a concern that does not allow another person to determine their direction. Several caregivers, in the different studies referred (Acton & Miller, 2003; Albinsson & Strang, 2003; Che et al., 2006), pointed out that living with their older relative has brought the opportunity of assuming their self‐care in the existential sense, which refers to the cure itself, that is, to open up themselves to the existential dimension, theirs and the other's; they are being in the world; that is facticity (Heidegger, 1962/1997; Moreira & Sales, 2010). The hermeneutics of facticity points to the fact that the person can only be understood in its own manner of being in the world, that is being in its factical life that allows it to be its existence (Heidegger, 1923/1999).

### 3.1 Limitations

Philosophical, theoretical, and scientific literature were used in this concept analysis and the concept facticity is sparse used outside the philosophical sphere. This could mean that the literature review was not thorough enough. It could also mean that scientific literature is presenting facticity, but not naming it as such. Another weakness could also be that no nursing text books were reviewed, but to our knowledge philosophical concepts are sparse in this literature. Another limitation could be the use of the concept analysis method by Walker and Avant (1995, 2005). We motivated our choice with the statement of Bergdahl and Berterö (2016) that important concepts can have many possible meanings and can be used in different ways in a discipline. That why it is important to clarify a philosophical concept and apply it in nursing dementia care.

### 3.2 Conclusion/Implications for nursing

Discussing implications for clinical practice means discussing the facticity of care. Nurses need to be aware that all human beings are not living in reality but with facticity. Being aware of facticity, nurses and healthcare providers may use creativity and search for understanding about how patients and next of kin perceive facticity. Even though there are limits inside the framework of everyday life, the framework is always broader than expected. When the nurse is open to the opportunities of integral care, she can promote connections between the different realities: nurse–patient and next of kin. Understanding facticity strengthens the understanding of our realities and thereby also improves the quality of care.

The use of this definition and application of the concept facticity should be tested and researched. Further research should focus on how to apply facticity in all types of caring encounters. Facticity is often applied in different situations, but it seems as if nurses and healthcare providers are not aware of this. Facticity means that we are exposed to different life situations and we interact and act in different ways due to these situations. Sometimes are we actively attending, other times are we giving up. Emotional are we in different modes; compassionate, emphatic or even ignorant. These ways of being are fundamental as human beings and as caring.

## CONFLICT OF INTEREST

The authors have declared no conflict of interest.

## AUTHOR CONTRIBUTIONS

All authors have contributed to the design, the data collection, data analysis, and writing of the manuscript.
